# Association of oral health related subjective quality of life and severity of negative symptoms of treatment-resistant schizophrenia: a cross-sectional study in Croatia

**DOI:** 10.1192/j.eurpsy.2024.385

**Published:** 2024-08-27

**Authors:** N. Mimica, I. Pupić, K. Bosak, P. Folnegović Grošić, Ž. Bajić, I. Filipčić, V. Grošić, Z. Zoričić

**Affiliations:** ^1^Women’s Psychiatric Ward, Institute for Biological Psychiatry and Social Rehabilitation, Psychiatric Clinic Sveti Ivan; ^2^Children’s Hospital Zagreb; ^3^Psychiatric Clinic Sveti Ivan; ^4^Department of Psychiatry, University Hospital Centre Zagreb; ^5^ Research Unit “Dr. Mirko Grmek”, Psychiatric Clinic Sveti Ivan; ^6^University Department of Psychiatry, University Hospital Sestre Milosrdnice, Zagreb, Croatia

## Abstract

**Introduction:**

Patients diagnosed with schizophrenia, particularly those with severe negative symptoms (NS) and treatment resistant schizophrenia (TRS), have poorer oral health than the general population, which can have serious consequences beyond oral and dental problems, but remains poorly addressed in psychiatric clinical practice and mental health research.

**Objectives:**

To investigate the association between oral health-related subjective quality of life (OHR-sQoL) and severity of NS in TRS.

**Methods:**

We conducted a cross-sectional study in a tertiary psychiatric clinic in Croatia during 2022-2023. The target population were patients diagnosed with TRS with more pronounced NS. The outcome was the Self-Evaluation of Negative Symptoms (SNS) scale and its five dimensions. Exposure was OHR-sQoL measured by the Oral Health Impact Profile questionnaire (OHR-sQoL). We tested the hypothesis using multivariable linear hierarchical regression analysis.

**Results:**

We enrolled 130 participants with a median (interquartile range) age of 43 (36-51) years, with an equal number of women and men. Total SNS and OHR-sQoL scores were found to be significantly associated in both bivariate and multivariable analysis adjusted for a large number of covariates (R^2^ increase over the effect of covariates = 0.22; p < 0.001; false discovery rate < 5%). Total SNS score was significantly associated with the functional limitation dimension of the OHIP-49, as well as diminished emotional range with psychological discomfort, physical and psychological disability, and anhedonia with functional limitation.

**
Figure 1.** Scatter plot of the correlation between the total score of the Self-evaluation of Negative Symptoms (SNS) and the Oral Health Impact Profile (OHIP-49); the solid line represents the 80% smoothed local polynomial regression curve; the dashed line represents the linear regression line (n = 130)

**Image:**

**Figure:**
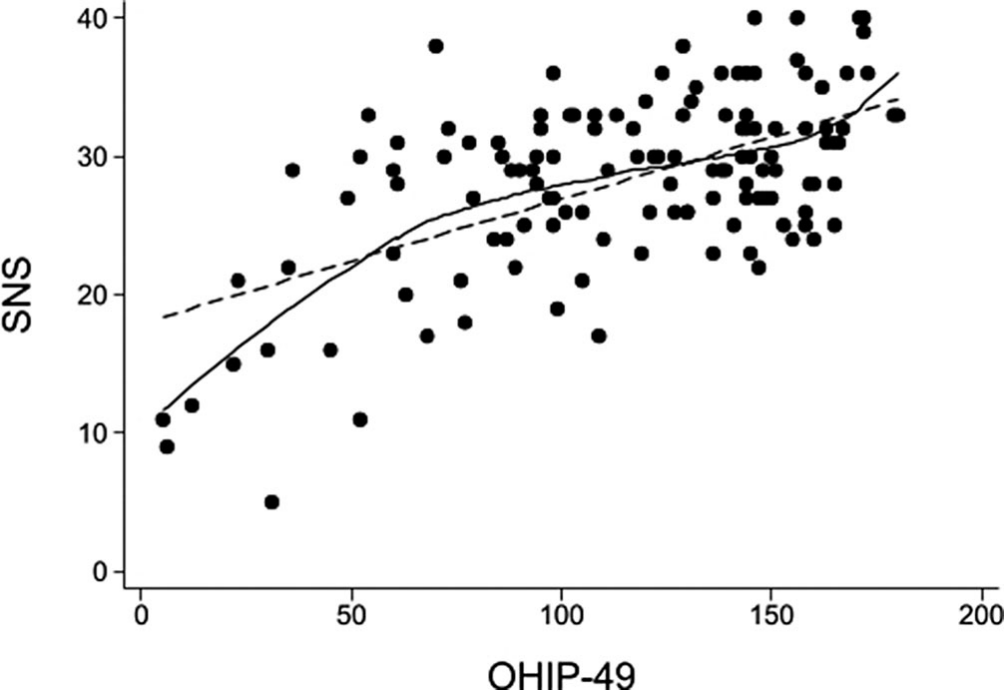

**Conclusions:**

NS of TRS are relatively strongly associated with OHR-sQoL, especially with functional limitations. The robustness of this association was confirmed by controlling for a large number of covariates. If the relationship between NS and OHR-sQoL is bidirectional, which should be verified by future studies, perhaps for further progress in solving the serious problems of NS and TRS it will be necessary to include the comorbidity with oral diseases and oral functional disorders and OHR-sQoL.

**Disclosure of Interest:**

None Declared

